# A dual-functional Janus nanofibrous membrane as an immunomodulatory barrier for periodontitis regeneration under diabetic conditions

**DOI:** 10.1016/j.mtbio.2026.102869

**Published:** 2026-02-05

**Authors:** Feiyang Wang, Yue Wang, Jiaqi Sheng, Kewei Zhang, Yu Cao, Xiang Han, Ke Yan, Xiaoqian Wang

**Affiliations:** a Department of Periodontology, The Affiliated Stomatological Hospital of Nanjing Medical University, Nanjing, 210029, People's Republic of China; b Jiangsu Province Key Laboratory of Oral Diseases, Nanjing, 210029, People's Republic of China; c Jiangsu Province Engineering Research Center of Stomatological Translational Medicine, Nanjing, 210029, People's Republic of China

**Keywords:** Janus membrane, Diabetes, Periodontitis, Alveolar bone loss

## Abstract

Periodontitis and diabetes mellitus exhibit a well-established bidirectional relationship, creating a hostile microenvironment characterized by persistent inflammation, oxidative stress, and impaired osteogenesis. Conventional guided tissue regeneration (GTR) membranes often yield suboptimal regenerative outcomes under these diabetic conditions due to their passive, monolithic structure. To address this limitation, we developed a novel dual-functional bilayer nanofibrous membrane, termed Pn@Janus TPP, through rational structural design specifically tailored for diabetic periodontitis. This Janus membrane features an anisotropic architecture: a dense barrier layer effectively blocks the infiltration of fast-proliferating soft tissue cells, while an opposite porous layer is functionalized with a polyethylene glycol (PEG) hydrogel incorporated with nano-hydroxyapatite (nHA) to enhance hydrophilicity, sustained Ca^2+^ release, and osteoconductivity. Critically, the integration of tea polyphenol-functionalized graphene oxide (TPG) provides potent reactive oxygen species (ROS)-scavenging capacity, effectively mitigating the exacerbated oxidative stress characteristic of the diabetic periodontitis milieu. Under AGE (100 μg/mL) and LPS (100 ng/mL) conditions in vitro, the membrane significantly promoted the adhesion and osteogenic/cementogenic differentiation of bone marrow mesenchymal stem cells (BMSCs), while concurrently exhibiting potent ROS-scavenging capacity. In vivo, Pn@Janus TPP implantation markedly enhanced alveolar bone regeneration in a diabetic rat periodontitis model, restored periodontal architecture, and reduced the expression of key pro-inflammatory cytokines (IL-6, TNF-α, iNOS, IL-1β), without inducing systemic toxicity. Transcriptomic and molecular analyses revealed that the therapeutic effects were mediated, at least in part, through the suppression of the IL-17/TRAF-6/NF-κB signaling axis. The innovative Janus structure, combining spatially resolved physical barrier function with bioactive immunomodulation and osteogenesis promotion, positions Pn@Janus TPP as a promising advanced biomaterial for managing the complex regenerative demands of diabetic periodontitis.

## Highlights

• Janus nanofibrous membrane (Pn@Janus TPP) combines a dense barrier layer with a porous hydrogel-functionalized layer for anisotropic functionality.
• The membrane promotes osteogenic/cementogenic differentiation of BMSCs under high-glucose and inflammatory conditions.
• In vivo, Pn@Janus TPP enhances alveolar bone regeneration and inhibits the IL-17/TRAF-6/NF-kB signaling pathway.

## Graphical abstract

**Figure:**
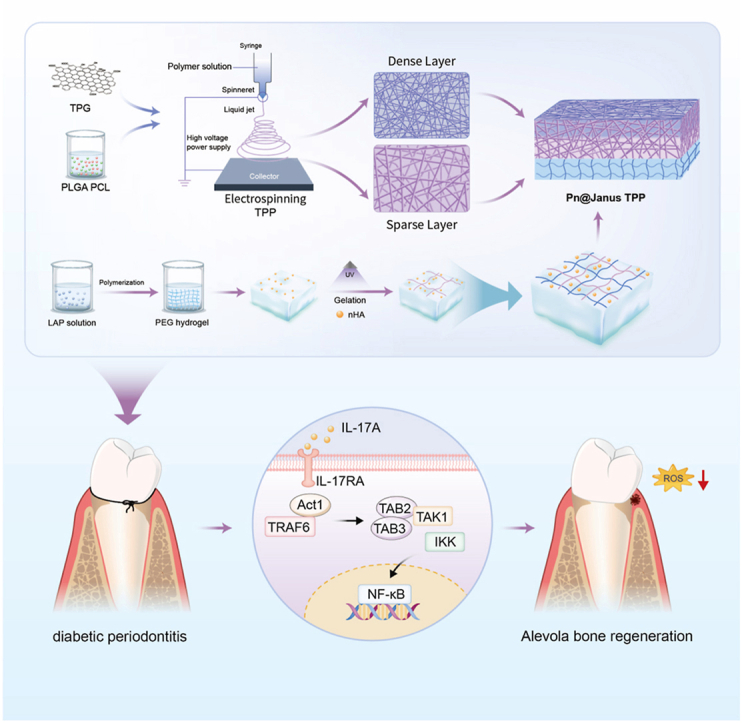

## 1 Background

Periodontitis is an inflammatory disease affecting the periodontal tissues, including the gingiva, periodontal ligament, and alveolar bone, and is characterized by gingival inflammation, loss of periodontal attachment, and destruction of surrounding structures [1,2]. As the sixth major complication of diabetes [3], periodontitis shares a well-established bidirectional relationship with diabetes mellitus [[4], [5], [6]]. Diabetes elevates the risk of periodontitis through mechanisms such as chronic inflammation and oxidative stress, while periodontal disease, in turn, exacerbates glycemic dyscontrol and metabolic complications via systemic inflammatory responses [[7], [8], [9], [10], [11]]. Consequently, controlling periodontitis is crucial for improving overall health in diabetic patients. Although the exact mechanisms linking periodontitis to diabetes remain incompletely understood, low-grade systemic inflammation is recognized as a key contributing factor [12,13]. Guided tissue regeneration (GTR) has been widely adopted in periodontal therapy to promote the regeneration of periodontal structures by using barrier membranes to exclude epithelial and connective tissue in growth, thereby facilitating repopulation by periodontal ligament-derived cells [[14], [15], [16]]. However, conventional GTR membranes, which primarily function as passive physical barriers, lack the ability to address the distinct biological requirements of the soft tissue interface versus the hard tissue defect site, and they lack the bioactivity to modulate the pathological diabetic microenvironment [17,18].

To overcome these limitations, the concept of Janus or bilayer membranes has emerged as a promising strategy [19,20]. This design philosophy recognizes that the ideal barrier should possess anisotropic properties: one side should be dense and smooth to effectively block the invasion of fast-proliferating soft tissue cells, while the opposite side should be porous and bioactive to promote the recruitment, adhesion, and osteogenic differentiation of progenitor cells [21]. Such a bifunctional design mimics the native periodontal tissue interface, providing spatial control over cell behavior [[22], [23], [24]].

In the realm of material innovation, addressing the complex inflammatory microenvironment associated with periodontitis with diabetes requires the development of materials capable of shielding stem cells from pro-inflammatory mediators, thereby preserving their viability and differentiation potential. Tea polyphenols (TPs), a class of natural antioxidants, are notable for their potent ROS scavenging capabilities. Specifically, epigallocatechin gallate (EGCG), a key component of TPs, has been demonstrated to significantly eliminate ROS, suppress intracellular ROS generation, modulate immune cell functions, and inhibit autoimmune inflammation. Leveraging these properties, we utilized TPs to reduce and functionalize graphene oxide (GO), resulting in the synthesis of a novel nanomaterial: tea polyphenol-functionalized graphene oxide (TPG). The reinforced barrier function of the material, also impeding the infiltration of inflammatory cells and the diffusion of inflammatory cytokines. PEG based hydrogels, particularly those constructed from 4-arm PEG-acrylate and poly(lactic-co-glycolic acid) (PLGA), exhibit biocompatibility and controlled in vivo degradability, and have received FDA approval for biomedical applications [25]. Their inherent hydrophilicity promotes cell adhesion, proliferation, and differentiation. Furthermore, the porous architecture of PEG hydrogels facilitates the free diffusion of oxygen, nutrients, and metabolic waste, while providing a conducive environment for cell migration and tissue integration [26]. The mechanical properties, such as strength and elasticity, can be precisely tailored to meet requirements. Additionally, these hydrogels serve as effective carriers for various bioactive molecules, enabling sustained release and prolonged therapeutic effects. The PEG segments contribute to a reduction in inflammatory cell infiltration through a combination of hydrophilic interactions and steric hindrance [27]. Studies have indicated that PEG-modified PLGA/poly(ε-caprolactone) (PCL) materials can form a physical barrier in vivo [28,29], obstructing the migration of fibroblasts and immune cells to the injury site. This mechanism helps delay the progression of the inflammation-bone resorption vicious cycle often observed in diabetic periodontitis. Moreover, PEG modification enhances the in vivo retention time of the material [30], supporting sustained drug release and prolonged anti-inflammatory activity.This crosslinked PEG hydrogel network serves not only as a carrier for nHA but also creates a hydrated, three-dimensional microenvironment that regulates the diffusion of nutrients and signaling molecules while providing tunable physical cues that can influence cell behavior.

Our study developed a novel bilayer barrier membrane, termed PEG/nHA@Janus 0.5%TPG/PLGA/PCL (abbreviated as Pn@Janus TPP), through rational design and functionalization. This membrane represents an upgrade from our previously developed TPP membrane (composed of TPG, PLGA, and PCL) [31], which already demonstrated good biocompatibility and regenerative potential. However, the fiber membrane was found to have less than ideal hydrophilicity and inadequate cell adhesion. By optimizing the electrospinning process, we engineered a Janus-type nanofibrous scaffold with a dual-sided structure: one side is dense and smooth, serving as a physical barrier against fibroblast infiltration, while the opposite side is porous and further modified with PEG hydrogel incorporated with nHA to enhance osteoconductivity, bioactivity, and cellular adhesion [32].

This investigation systematically evaluates the physicochemical properties, biocompatibility, and dual functionality of the upgraded Pn@Janus TPP membrane—both in vitro under high-glucose and inflammatory conditions, and in vivo using a diabetic rat model of periodontitis. We hypothesize that this advanced membrane can not only act as a physical barrier but also modulate the local inflammatory response, reduce oxidative stress, and promote osteogenic/cementogenic differentiation, thereby facilitating effective periodontal regeneration under diabetic conditions.

In this study, we propose a novel electrospun-hydrogel integrated Janus membrane that not only offers new perspectives for the design of multifunctional barrier membranes, but also presents a promising therapeutic strategy for enhancing bone regeneration in periodontitis complicated by diabetes mellitus.

## 2 Materials and methods

### 2.1 Preparation of Pn@Janus TPP fibrous membranes

PLGA (LA:GA = 50:50, MW = 106000, Jinan Daigang Biomaterial) and PCL (80000 kD, Aladdin) were dissolved in HFIP (Aladdin) respectively, and the two solutions were pre-mixed at a volume ratio of 50:50, with acetic acid added (volume ratio of acetic acid to HFIP was 0.2%) to adjust the solution properties. TPG (tea polyphenols functional graphene oxide, Jiangsu Xianfeng Nanomaterial Technology) was dispersed in the above PLGA/PCL mixed solution to prepare a 10% (w/v) polymer solution with a TPG mass fraction of 0.5%. The mixed solution was thoroughly stirred using an ultrasonic device and a magnetic stirrer at room temperature for 24 h to ensure uniform dispersion of each component.

The prepared Janus TPP mixed solution was loaded into a 10 mL syringe equipped with a 16G injection needle, which was fixed on an electrospinning device (JDF05, Nayi, China). The electrospinning environment was set with a relative humidity of 50% and a temperature of 25 °C. A micro-injection pump was started, the solution flow rate was adjusted to 1 mL/h, a high-voltage DC power supply was set to 15 kV, and the distance between the syringe needle and the collector (covered with 20 cm × 20 cm smooth silicone paper) was maintained at 20 cm. Electrospinning was carried out continuously; when half of the spinning solution in the glass syringe remained, the 16G injection needle was replaced with a 22G one, and the voltage of the high-voltage DC power supply was adjusted to 25 kV, with electrospinning continued until completion. After spinning, the collected fiber membrane was vacuum-dried at room temperature for 3 days to completely remove residual organic solvents, obtaining a Janus TPP membrane, with the two sides designated as sparse TPP and dense TPP according to differences in porosity and fiber diameter.

To prepare the hydrogel, 20 mg LAP was added to 1 mL pure water to prepare a 20 mg/mL LAP mother solution, which was fully vortexed and heated in a 40 °C water bath for 15 min to dissolve. Appropriate PEG powder (4-arm, 20000 kD, Sinopeg, China) was added to pure water to prepare a 100 mg/mL PEG solution, and the above LAP mother solution was diluted with the PEG solution to ensure the final LAP concentration was 1 mg/mL nHA(nano-Hydroxylapatite) (Jiangsu Xianfeng Nanomaterial Technology) was added to the solution to a concentration of 15 mg/mL, which was fully vortexed and heated in a 40 °C water bath for 20 min to obtain a uniform hydrogel liquid. The prepared hydrogel liquid was evenly spread on the sparse surface of the electrospun Janus TPP nanofiber membrane, gently flattened with a glass plate to ensure full contact and uniform coverage, and then irradiated with a 405 nm light source to induce gelation, firmly fixing the hydrogel on the fiber membrane surface. The final composite membrane was Pn@Janus TPP.

### 2.2 Physicochemical characterization of the Pn@Janus TPP

The morphological traits and structural features of the Pn@Janus TPP nanofibrous barrier membrane were examined under a scanning electron microscope (SEM, Hitachi, Japan) at a voltage of 15 kV. Prior to observation, the composite membrane of PEG/nHA hydrogel and Janus TPP nanofibrous barrier membrane was placed in liquid nitrogen for 1 h, then stored in a −80 °C refrigerator overnight, and subsequently freeze-dried using a freeze dryer (Xinzhi Biology, China). The fiber diameter of the SEM images was measured using Image J software, and the porosity was analyzed using Matlab software.

The hydrophilicity of the membrane was evaluated through static water contact angle measurement (Automatic Contact Angle Meter Model SL200B, China). The material was fixed on a holder, a 10 μL drop of deionized water was dropped on the surface, and the contact angle of the fiber membrane was measured. The average value of 5 measurement points was taken as the final result.

The mechanical properties of the membrane were tested by a universal testing machine (DZF-6020, China) at room temperature. The prepared materials were cut into strips with no defects or flaws. The two ends of the material were clamped with fixtures and fixed on the universal testing machine. A force of 100 N was applied to stretch the material at a constant speed of 5 mm/min until it broke. Each material was tested 3 times, and stress-strain curves were recorded to statistically analyze the tensile strength, breaking strain, and Young's modulus.

The Ca^2+^ release behavior of the membrane was determined by measuring the Ca^2+^ concentration in PBS solution on days 1 to 16. The test materials were cut into 1 cm^2^ pieces, cleaned with deionized water to remove surface impurities, and then placed in labeled centrifuge tubes. An appropriate amount of PBS solution was added to each tube to completely immerse the material. The centrifuge tubes were placed on a 37 °C constant temperature shaker with an oscillation frequency of 120 rpm, and samples were taken daily. According to the instructions of the calcium ion detection kit, the absorbance was measured at 610 nm using a microplate reader (Spectramax 190, MD, USA), and the Ca^2+^ concentration was calculated through a standard curve. The cumulative release percentage at each time point was calculated as [(C_t_ - C_0_) × V/(C_0_ × V)] × 100% (where C_0_ is the initial calcium ion concentration, C_t_ is the calcium ion concentration at time t, and V is the system volume).

The in vitro degradation profile of the Pn@Janus TPP membrane was evaluated by monitoring the remaining weight over time. Briefly, pre-weighed membranes (n = 3) were immersed in 10 mL of phosphate-buffered saline (PBS, pH 7.4) and incubated at 37 °C under gentle agitation. At predetermined time points the samples were retrieved, thoroughly rinsed with deionized water, and lyophilized to constant weight. The remaining weight percentage was calculated using the formula: Remaining Weight (%) = (W_d/W_0) × 100, where W_0 is the initial dry weight and W_d is the dry weight after degradation.

### 2.3 Cell culture

Bone marrow mesenchymal stem cells (BMSCs) and Human Gingival Fibroblasts (HGFs).were provided by Jiangsu Province Key Laboratory of Oral Diseases. They were cultured in α-MEM medium containing 10% FBS and 1% penicillin-streptomycin solution in an incubator (37 °C, 5% CO_2_).

### 2.4 Cytocompatibility of the fibrous membranes

The BMSCs viability assay was performed using a live/dead staining kit (Dojindo, Japan). BMSCs were seeded into 12-well plates and co-cultured with different materials. After 24 h and 72 h of co-culture, 2 mL of live/dead staining solution was added to each well, followed by incubation in a 37 °C, 5% CO_2_ incubator in the dark for 30 min. Live cells (green fluorescence) and dead cells (red fluorescence) were imaged via an inverted microscope (Leica, Germany). The red blood cell hemolysis test used SD rat blood. Blood was collected into heparinized tubes, diluted with PBS, and centrifuged at 2000 rpm for 10 min. The precipitate was washed with PBS and centrifuged repeatedly until the supernatant became clear, then 0.5 mL of the precipitate was mixed with 9.5 mL of sterile PBS buffer to prepare a red blood cell suspension. Pn@Janus TPP was added to 0.5 mL of the suspension, the volume was adjusted to 1 mL with PBS solution, and the mixture was gently pipetted to mix well. After standing at 37 °C for 4 h, it was centrifuged at 1500 rpm for 10 min. The supernatant was collected to measure ultraviolet-visible absorbance at 562 nm using a microplate reader, and the hemolysis rate (%) was calculated by the formula: [(A experimental group - A negative control group)/(A positive control group - A negative control group)] × 100%, with the pure water group as the positive control and the PBS group as the negative control.

### 2.5 Cell adhesion test on both sides of the polyurethane membranes

The adhesion property of BMSCs to the Janus nanofibrous barrier membrane was tested by the method of standard live/dead staining assay (Dojindo, Japan). Briefly, the Janus TPP nanofibrous barrier membrane was cut into wafers and divided into two groups according to its surface characteristics: the dense (smooth) surface and the sparse (rough, hydrogel-combined) surface. The two groups of membranes were placed in a 12-well plate and subjected to UV sterilization. BMSCs were seeded onto the two surfaces at a density of 2 × 10^4^ cells/well respectively, and cultured in a 37 °C, 5% CO_2_ incubator for 48 h. After culture, the culture medium was discarded, and the membranes were washed gently with PBS to remove unadhered cells. Then, the cells were fixed with 4% paraformaldehyde for 30 min, permeabilized with 0.1% Triton X-100 (Beyotime, China) for 15 min, and blocked with 0.5% BSA for 1 h. The cytoskeleton was stained with rhodamine phalloidin (Beyotime, China) diluted with BSA (incubated in the dark for 30 min), and the nucleus was stained with DAPI (Beyotime, China) for 10 min. The cell adhesion morphology was observed using a laser scanning confocal microscope (LSM 800, Carl Zeiss, Germany), and the cell adhesion area was quantified using Image J software.

### 2.6 The regulation of membranes on BMSCs osteogenesis and cementogenesis

BMSCs were seeded onto TPP and Pn@Janus TPP membranes at a density of 2 × 10^5^ cells/well, cultured in complete medium at 37 °C with 5% CO_2_ for 24 h, then the medium was discarded, the wells were washed twice with PBS, and 2 mL of BMSCs-specific osteogenic induction medium was added to divide the cells into four groups: blank control group (Ctrl), inflammation group (100 μg/mL AGE + 100 ng/mL LPS), TPP group (100 μg/mL AGE + 100 ng/mL LPS), and Pn@Janus TPP group (100 μg/mL AGE + 100 ng/mL LPS), with medium changed every 3 days for continuous culture (14 days for gene/protein detection, 28 days for mineralization detection). For qRT-PCR, after 14 days of culture, cells were lysed with Trizol to extract RNA, which was reverse transcribed into cDNA (500 ng RNA, 2 μL 5 × PrimeScript RT Master Mix, enzyme-free water to 10 μL; program: 37 °C 15min, 85 °C 5s, 4 °C 5min), then qRT-PCR was performed with a 20 μL system (10 μL 5 × PrimeScript RT Master Mix, 0.8 μL forward/reverse primer, 1.6 μL cDNA, 6.8 μL RNase Free dH_2_O; program: 95 °C 3min, 40 cycles of 95 °C 10s/60 °C 1min, 65 °C 5s/95 °C 5s) to detect osteogenic (Ocn, RUNX2, Alp, Opn) and cementogenic (Cemp-1) genes with GAPDH as internal reference. The sequences for all primers used are listed in Table 1. For Western blots, after 14 days of culture, cells were lysed with RIPA containing 1% PMSF, protein concentration was determined by BCA method, protein samples were separated by SDS-PAGE, transferred to PVDF membrane, blocked with 5% skimmed milk, incubated with primary antibodies (ALP, Abcam #ab229126; CEMP-1, Abcam #ab134231; OCN, Proteintech #16157-1-AP; OPN, Proteintech #30200-1-AP; RUNX2, Proteintech #207001-1-AP; ACTIN, Proteintech #66009-1-Ig) at 4 °C overnight, then with secondary antibodies, and developed for protein detection. For ALP staining/activity determination (14 days), cells were fixed with 4% paraformaldehyde, stained with BCIP/NBT(Beyotime, China) working solution, and ALP activity was calculated by measuring absorbance at 520 nm; for alizarin red staining/quantitative analysis (28 days), cells were stained with alizarin red, calcium nodules were dissolved with 10% CPC, and calcium content was quantified by absorbance at 595 nm. Based on these results, the fibrous membrane with 0.5% TPG (optimal concentration) was selected for subsequent studies.

**Table 1:** Primer sequences for qRT-PCR.

Gene	Forward Primer(5′-3′)	Reverse Primer(5′-3′)
*Gapdh*	ACGGGAAACCCATCACCATC	TCACAAACATGGGGGCATCA
*Ocn*	GCAGGAGGGCAGTAAGGTG	AAGCCAATGTGGTCCGCTA
*RUNX2*	CACCCAGTAGCAAACCGAAAC	TGTAAATGCTGCTGAAATAGGC
*Alp*	GGACGGTGAACGGGAGAA	CGTGAAGCAGGTGAGCCAT
*Cemp-1*	ATGGCGCTCAAGAAGATCCA	TCACTGGGTTGCTGCTGTAG
*Opn*	ATTCTCGAGGAAGCCAGCCA	AGTGTTTGCTGTAATGCGCC
*Mkk3*	AGAGGGGGATGTGTGGATCT	CTTGCTGTGTAGGTGCTCCA
*NF-kb*	ATCAAAGAGCTGGTGGAGGC	TGTGGAGGAGGACGAGAGAG
*P38*	GCCCCCGAGATTATGCTGAA	TCCCCGTCAGACGCATTATC
*IL-17α*	CAAACGCCGAGGCCAATAAC	AGGGTGAAGTGGAACGGTTG
*IL-17f*	AGGAAGACGGCTCCATGAAC	GTGCAGCCAACTTTTATGAGCA
*TNF-α*	ATCCGAGATGTGGAACTGGC	CGATCACCCCGAAGTTCAGT
*TRAF-6*	CTCAGCGCTGTGCAAACTAC	TGATCAAGGATCGTGAGGCG

### 2.7 RNA sequencing and bioinformatics analysis

Total RNA was extracted using TRIzol reagent and sequenced by cosmos wisdom. Differential gene expression analysis was performed with edgeR (|logFC| ≥ 2, padj <0.05), and results were visualized via heatmaps/Venn diagrams (https://www.bioinformatics.com.cn.). Functional enrichment (GO/KEGG, P < 0.05) was conducted using clusterProfiler, while GSEA (MSigDB C2/C5; FDR <0.05) identified key pathways.

### 2.8 In vivo treatment

Female SD rats (6–8 weeks old) were randomly allocated into a two-stage experiment. In the first stage, rats were divided into four groups: healthy control (Healthy, n = 4), diabetes only (DB, n = 4), periodontitis only (PD, n = 4), and diabetic periodontitis (DP, n = 16). Type 2 diabetes was induced by administering a high-fat diet for two weeks followed by intraperitoneal injections of streptozotocin (STZ, 30 mg/kg in citrate buffer) every three days; animals with persistent random blood glucose levels ≥16.7 mmol/L were considered diabetic. Experimental periodontitis was then induced in the PD and DP groups by ligating the left maxillary second molars with 4-0 silk sutures for 14 days. In the second stage, the remaining DP rats (n = 12) were randomly assigned to three treatment groups (n = 4 per group): a DP control group (no implant), a group implanted with the TPP membrane, and a group implanted with the Pn@Janus TPP membrane. Under anesthesia, a full-thickness flap was elevated from the first to the third molar, and the respective membranes were positioned to completely cover the periodontal defect, extending 1 mm beyond its margins, before suturing and securing with a periodontal pack. Body weight and blood glucose levels were monitored throughout the 28-day study period, after which all rats were euthanized, and maxillae were collected and fixed in 4% paraformaldehyde for subsequent micro-CT and histological analysis.

### 2.9 Micro-CT evaluation

The harvested maxillae were scanned using a high-resolution micro-CT system (Skyscan 1176, Bruker, Belgium). The acquired images were reconstructed into three-dimensional (3D) models using NRecon software (Version 1.6). Quantitative analysis was performed with CTAn software (Version 1.14). The BV/TV was measured within a defined region of interest (ROI) at the palatal furcation area of the second molar. Additionally, the distance from the alveolar ABC to TP, expressed as the ABC-TP/CEJ-TP ratio, was quantified to evaluate alveolar bone height changes.

### 2.10 Histology and immunofluorescence staining

The sections were subjected to H&E staining to assess general tissue morphology and periodontal attachment loss, and Masson's trichrome staining to evaluate collagen fiber organization and maturation. For immunohistochemical (IHC) analysis, sections were incubated with the following primary antibodies: OCN (Proteintech, 16157-1-AP), RUNX2 (Proteintech, 207001-1-AP), IL-6 (Proteintech,26404-1-AP), anti-TNF-α (Proteintech, 17590-1-AP), and anti-IL-1β (Proteintech,66737-1-lg).

### 2.11 Statistical analysis

Data from three independent replicates are expressed as mean ± standard deviation (SD). Differences between two groups were assessed by an unpaired Student's t-test, while comparisons among multiple groups were analyzed by one-way ANOVA with Tukey's post-hoc test using GraphPad Prism 7. Statistical significance is denoted as follows: ∗p < 0.05, ∗∗p < 0.01, ∗∗∗p < 0.001; ns, not significant.

## 3 Results

### 3.1 Characterization of the Janus fibrous membranes

**Fig. 1:**
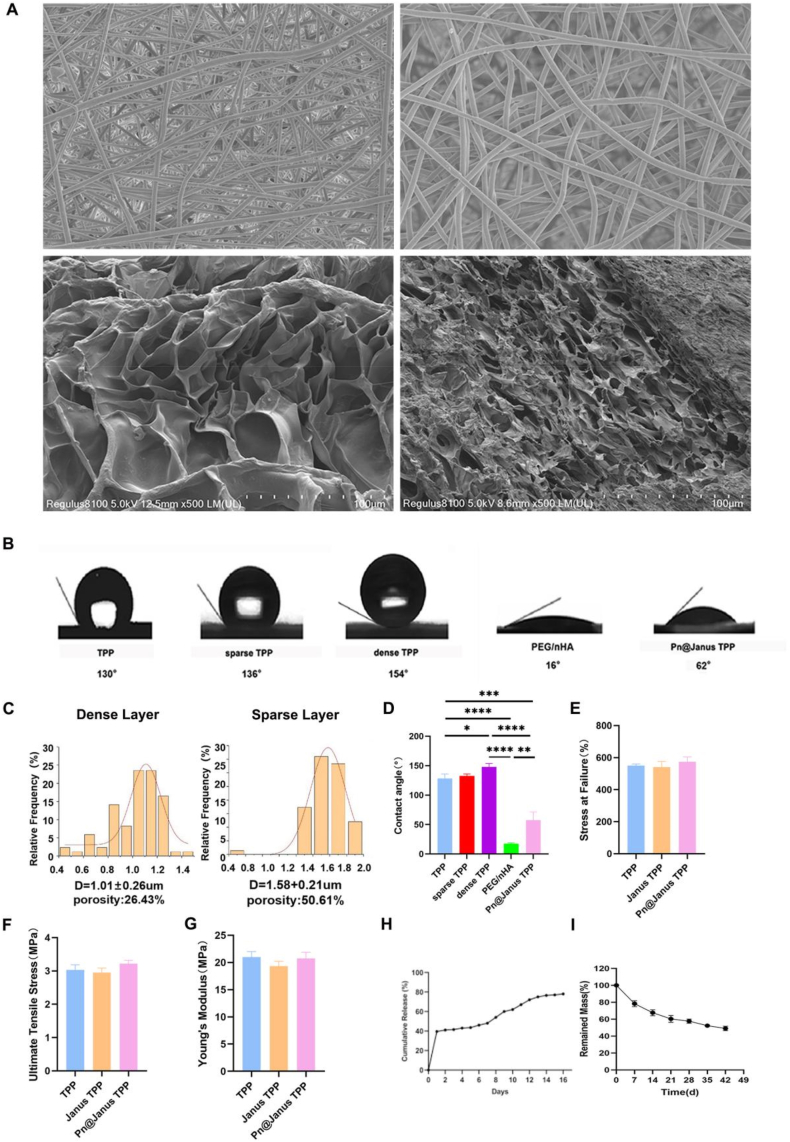
Characterization of the Pn@Janus TPP membrane. Structure and characterization of Pn@Janus TPP (A) SEM images of dual side of Pn@Janus TPP. (B) Schematic of the contact angle. (C)Fiber Distribution Quantification. (D)Contact angle measurement. (E)Stress at failure. Testing. (F)Ultimate tensile stress testing. (G)Young's modulus testing. (H)Cumulative Ca^2+^ion release profile over time. (I) In vitro degradation profile. Data are shown as mean ± SD and analyzed by one-way ANOVA with Tukey's multiple comparisons test. Statistical significance is set as ∗P < 0.05, ∗∗P < 0.01, ∗∗∗P < 0.001.

The fabrication process of Pn@Janus TPP nanofibrous barrier membrane is illustrated in graphic abstract. The morphology of the Pn@Janus TPP was characterized by Scanning Electron Microscopy (SEM) (Fig. 1A). One side of the membrane exhibited a porous structure (designated as sparse TPP), while the other side presented a dense barrier structure (designated as dense TPP). SEM analysis revealed that the dense TPP side had a fiber diameter of 1.01 μm and a porosity of 26.43%, whereas the sparse TPP side showed a larger fiber diameter of 1.57 μm and a higher porosity of approximately 50.61% (Fig. 1C). The porous PEG/nHA hydrogel conjugated to the sparse side formed a three-dimensional network structure, which not only served as a sustained-release platform for therapeutic agents but also created a favorable microenvironment for cell migration and proliferation.

The hydrophilicity of the materials, a crucial factor influencing cell adhesion and interaction, was evaluated by measuring the water contact angle (WCA) (Fig. 1B). The results demonstrated that the hydrophilicity of Pn@Janus TPP was significantly higher than that of the Janus TPP. The water contact angle of the Pn@Janus TPP was measured to be 62° (Fig. 1D). In contrast, the contact angles of the dense TPP and sparse TPP sides of the Janus TPP were 136° and 130°, respectively, indicating strong hydrophobicity. The pure PEG/nHA hydrogel exhibited the highest hydrophilicity with a contact angle of 16°, confirming that the incorporation of the hydrogel effectively improved the surface wettability of the Janus nanofibrous membrane.

Mechanical properties are essential for the clinical application of barrier membranes, as they determine the ability to maintain space and resist deformation during tissue regeneration. Tensile tests were conducted to evaluate the mechanical performance of the materials (Fig. 1E–G). The results showed that there were no significant differences in tensile strength and elastic modulus between Pn@Janus TPP, Janus TPP, and the single-layer TPP. This indicated that the conjugation of the PEG/nHA hydrogel did not compromise the mechanical integrity of the nanofibrous barrier membrane, ensuring its suitability for providing stable mechanical support in the dynamic oral environment.

The release behavior of calcium ions (Ca^2+^) from the Pn@Janus TPP was monitored over a 16-day period to assess its potential for promoting osteogenic mineralization (Fig. 1H). The calcium ion release profile exhibited a two-stage pattern: an initial rapid release phase (0–2 days) where the cumulative release amount quickly increased to 40%, followed by a sustained slow release phase. After 2 days, the release rate slowed down, and by day 14, the cumulative release rate approached 80%, after which the release process gradually stabilized. This biphasic release characteristic not only ensures the rapid availability of calcium ions in the early stage to initiate osteogenic activity but also provides a long-term, steady supply of ions to maintain the microenvironmental homeostasis required for bone regeneration.

Gravimetric analysis of the fibrous membranes in physiological solution revealed a significant weight loss during the first two weeks (Fig. 1I). Subsequently, the degradation rate of Pn@Janus TPP decelerated, entering a sustained, slower degradation phase from the third week onward.

### 3.2 Assessment of cell viability, barrier integrity, and cellular attachment on the Janus membrane

**Fig. 2:**
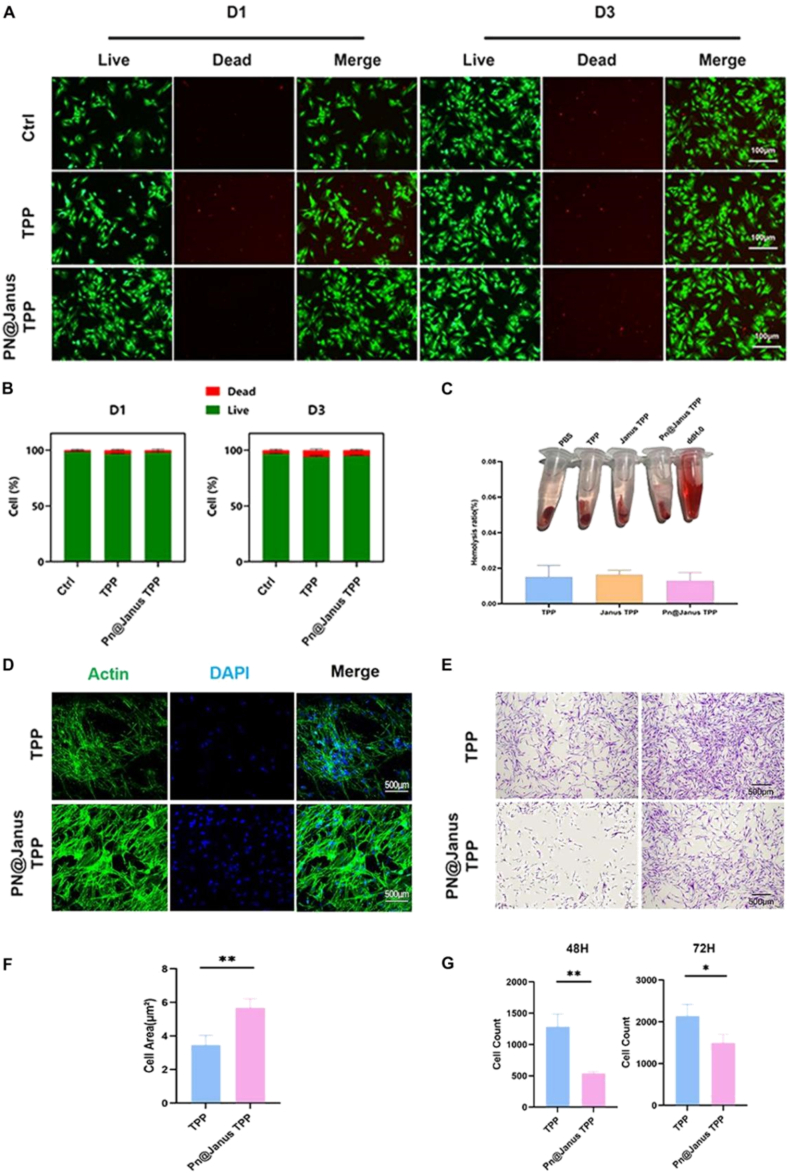
In vitro cytocompatibility, cell adhesion, and barrier function of the nanofibrous membranes.(A) Representative Live/Dead staining images of BMSCs co-cultured with different membranes for 24 and 72 h (green: live cells; red: dead cells). (B) Quantitative analysis of cell viability,showing no significant cytotoxicity across all groups.(C) Hemocompatibility assessment. Photographs of red blood cells after contact with materials and the corresponding quantitative hemolysis rates. All samples exhibited hemolysis rates below the 5% safety threshold, indicating excellent blood compatibility.(D) CLSM images of cytoskeletal organization in BMSCs adhered to the membrane surfaces after 48 h (F-actin: red; nuclei: blue). The Pn@Janus TPP group (porous side) promotes cell spreading and a well-organized cytoskeleton. (E) Representative images of Transwell assays evaluating the migration of Human Gingival Fibroblasts (HGFs) towards the dense side of the membranes after 48 and 72 h. Migrated cells were stained with crystal violet. (F) Quantitative analysis of cell adhesion area. The Pn@Janus TPP group showed a significantly larger cell spreading area compared to the TPP group. (G) Quantitative analysis of HGF migration. The dense side of Pn@Janus TPP significantly inhibited HGF migration compared to the control TPP membrane at both time points.Statistical significance is set as ∗P < 0.05, ∗∗P < 0.01, ∗∗∗P < 0.001.

Live/dead staining assays were performed to assess the viability of BMSCs co-cultured with different membrane samples at 24 h and 72 h (Fig. 2A). At 24 h, almost no red-fluorescent dead cells were observed in any group, indicating excellent initial cell compatibility. By 72 h, a slight increase in dead cells was noted across all groups, but there was no statistically significant difference between groups (Fig. 2B), demonstrating that the Janus membrane did not exert cytotoxic effects on BMSCs.

Biosafety is a critical criterion for biomedical materials intended for in vivo implantation. A red blood cell (RBC) hemolysis test was performed to evaluate the hemocompatibility of the Pn@Janus TPP, Janus TPP, and TPP membranes (Fig. 2C). The hemolysis rate of all membrane samples was below 5%, which meets the safety standard for biomedical implants. This result confirmed that the composite membrane did not induce RBC lysis, eliminating the risk of intravascular hemolytic reactions when used in clinical settings.

Cell adhesion behavior on the membrane surface is a prerequisite for subsequent cell proliferation and differentiation. Laser Scanning Confocal Microscopy (CLSM) was used to observe the adhesion and spreading of BMSCs on the Pn@Janus TPP and TPP membranes after 48 h of co-culture (Fig. 2D). The Pn@Janus TPP group (porous side with PEG/nHA hydrogel) showed extensive cell coverage, with BMSCs exhibiting an elongated, flattened morphology and anchoring to the membrane surface via numerous filopodia and lamellipodia. In contrast, only sparse, scattered single cells were observed on the surface of the TPP membrane. Quantitative analysis revealed that the average cell area in the Pn@Janus TPP group was approximately 1.8-fold higher than that in the TPP group (Fig. 2F), confirming the superior cell adhesion and interface compatibility of the Pn@Janus TPP.

To evaluate the barrier function of the dense side of the Janus membrane, Transwell assays were conducted to assess the migration of Human Gingival Fibroblasts (HGFs) (Fig. 2E). At 48 h, the number of migrated HGFs in the TPP group was more than twice that in the Pn@Janus TPP group. Even at 72 h, the TPP group still showed a significantly higher HGF migration count compared to the Pn@Janus TPP group (Fig. 2G). This result indicated that the dense side of the Pn@Janus TPP effectively inhibited the migration of HGFs, thereby preventing soft tissue invasion into the bone defect area and creating a closed space for osteogenic regeneration.

### 3.3 In vitro stem cell osteoinductive ability of the Pn@Janus TPP with potent ROS-scavenging capacity

The in vitro stem cell osteoinductive ability of the Pn@Janus TPP nanofibrous barrier membrane was evaluated by BMSCs under lipopolysaccharide (LPS) (100 ng/mL) + advanced glycation end products-bovine serum albumin (AGE) (100 μg/mL) stimulation, which simulates the high-glucose inflammatory microenvironment of diabetic periodontitis.

**Fig. 3:**
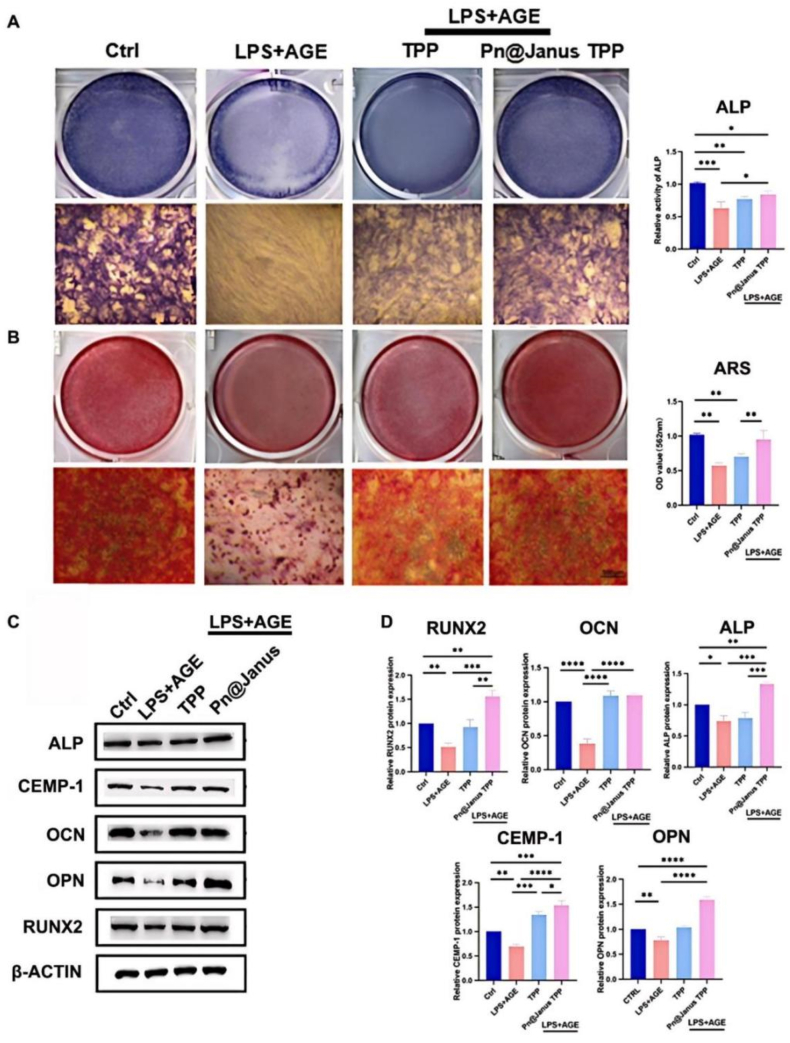
The regulation of PN@Janus TPP on BMSCs. (A)Alizarin red staining analysis of calcium content. (B) CPC quantitative analysis of calcium content. (C) Western blotting analysis ofBMSCsosteogenesis (ALP, RUNX-2, OPN, OCN), cementogenesis (CEMP-1) related protein expression. (D) Western blotting quantification Statistical significance is set as ∗P < 0.05, ∗∗P < 0.01,∗∗∗P < 0.001,∗∗∗∗P < 0.0001.

**Fig. 4:**
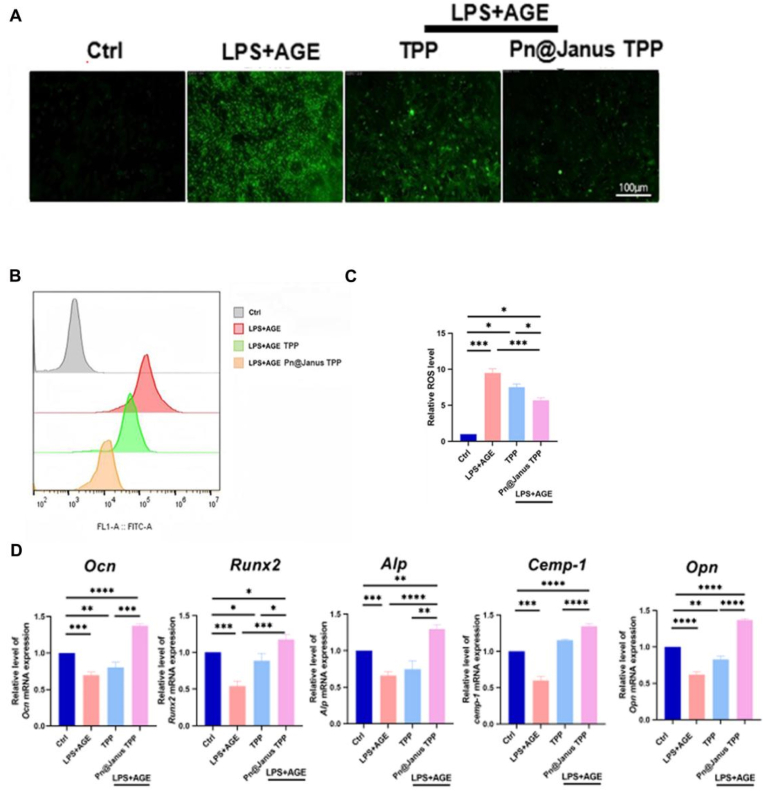
The regulation of PN@Janus TPP on BMSCs. (A) ROS staining images. (B) ROS fluorence. (C)ROS quantification. (D)qRT-PCR analysis of BMSCs osteogenesis (ALP,Runx-2, OPN, OCN), cementogenesis (CEMP-1). Statistical significance is set as ∗P < 0.05, ∗∗P < 0.01,∗∗∗P < 0.001,∗∗∗∗P < 0.0001.

After 14 days of osteogenic induction, quantitative real-time polymerase chain reaction (qPCR) results indicated that the mRNA expression levels of osteogenic markers (OCN, RUNX2, ALP, OPN) and the cementogenic marker (CEMP-1) in the LPS + AGE group were significantly downregulated compared with the control group (Fig. 4D). In contrast, the Pn@Janus TPP group exhibited a more prominent upregulation of these markers than the TPP group; specifically, the mRNA levels of late osteogenic markers OCN and OPN in the Pn@Janus TPP group were approximately twice those of the TPP group. Western blot analysis further confirmed this conclusion. For early osteogenic mineralization capacity (Fig. 3C), ALP staining and activity detection after 14 days of co-culture showed that the AGE + LPS group had weak ALP staining with sparse light blue precipitation, while the Pn@Janus TPP group presented dense dark blue staining areas, and its ALP activity was significantly enhanced, reversing the inhibitory effect of high-glucose inflammation on ALP activity (Fig. 3A). This was also evidenced by Alizarin Red S staining results (Fig. 3B).

The Pn@Janus TPP membrane significantly attenuated intracellular ROS in BMSCs under diabetic-inflammatory conditions, as evidenced by a marked reduction in fluorescence compared to the LPS + AGE group. This potent antioxidant effect is primarily attributed to the TPG component.

### 3.4 Mechanistic evidence of Pn@Janus-mediated IL-17 pathway inhibition in BMSCs

**Fig. 5:**
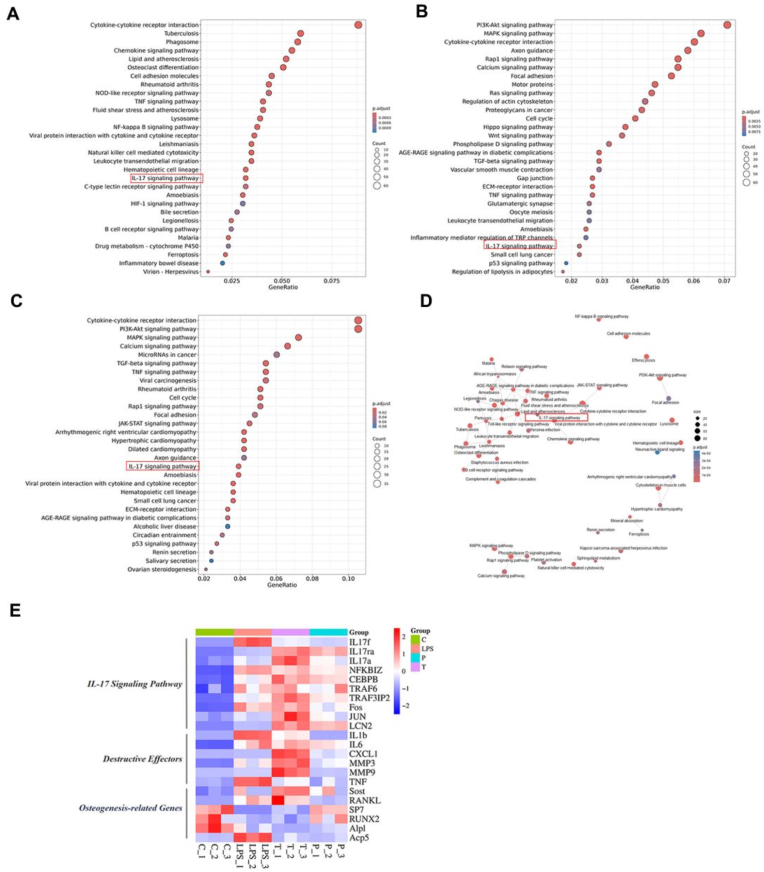
Transcriptomic analysis reveals Pn@Janus TPP suppresses the IL-17 signaling pathway to ameliorate the diabetic inflammatory microenvironment. (A-C) KEGG pathway enrichment analysis of the DEGs, highlighting the IL-17 signaling pathway as the most significantly enriched. (D) Gene-pathway interaction network. (E)Volcano plot of DEGs.

Based on our analysis, we demonstrate that the Pn@Janus composite hydrogel membrane exerts anti-inflammatory and pro-regenerative effects primarily through the precise inhibition of the IL-17 signaling pathway (Fig. 5A). RNA-seq and KEGG enrichment analysis pinpointed the IL-17 pathway as a central hub, intricately connecting anti-inflammatory, anti-osteoclastogenic, and anti-apoptotic processes (Fig. 5A–D). Central to the diabetes and periodontitis pathology is the IL-17 signaling pathway, which promotes neutrophil recruitment, osteoclast activation, and pyroptosis via TRAF6-mediated NF-κB activation. Pn@Janus TPP can selectively inhibit IL-17 signaling and downstream TRAF6/NF-κB activation. By targeting this pathway, Pn@Janus TPP not only mitigates periodontal inflammation but also addresses the diabetic exacerbation of oral bone loss.

As illustrated by the KEGG enrichment analysis (Fig. 5A–C), the significant enrichment of the “cytokine-cytokine receptor interaction” pathway suggests a plausible mechanism underlying the inflammatory exacerbation in the high-glucose inflammatory microenvironment (LPS + AGE). Under such conditions, BMSCs and surrounding immune cells perceive pathogen-associated molecular patterns and metabolic stress signals, leading to the upregulation of cytokine receptors such as IL-17R. The Pn@Janus TPP membrane demonstrates a superior capacity to disrupt this inflammatory cascade.

**Fig. 6:**
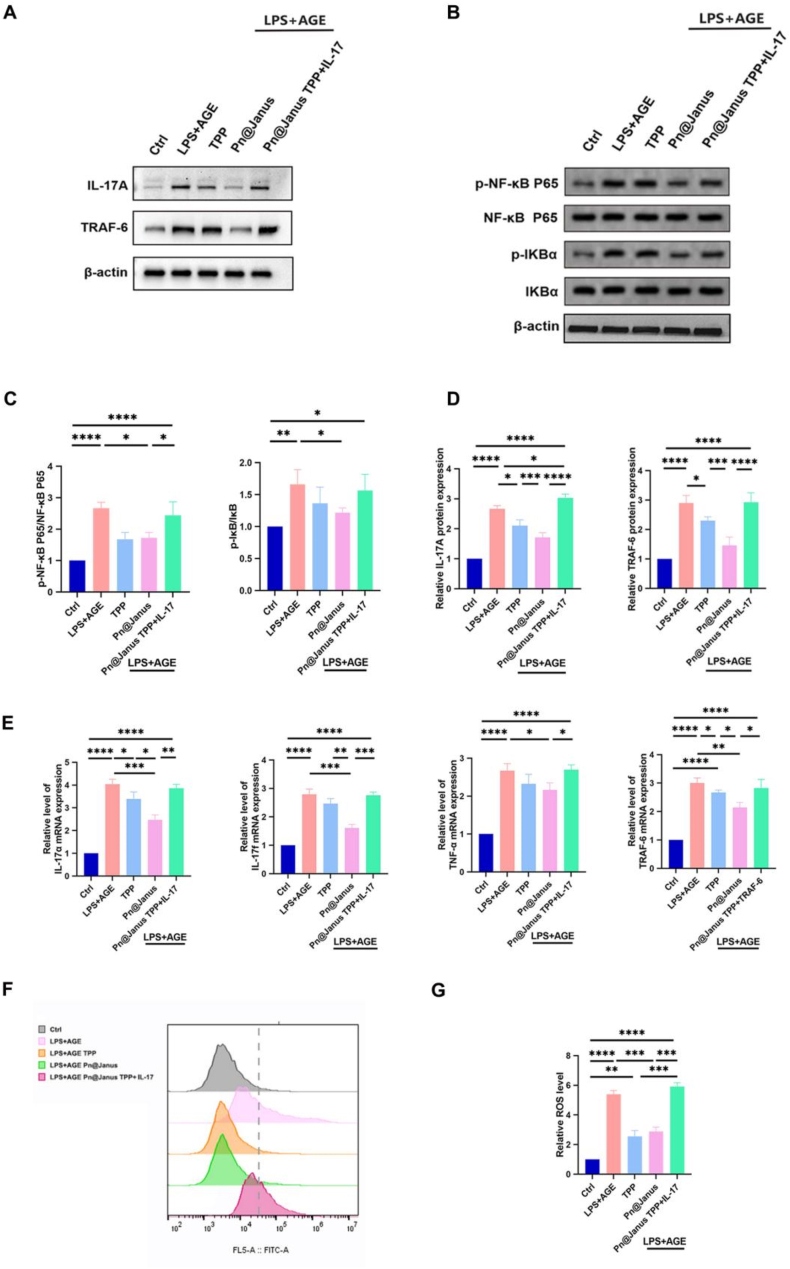
Transcriptomic analysis reveals Pn@Janus TPP suppresses the IL-17 signaling pathway to ameliorate the diabetic inflammatory microenvironment. (A)Western blot analysis showing inhibition of IL-17 signaling and TRAF6. (B)Western blot analysis of NF-κB activation. (C) Western blot quantification of IL-17 signaling pathway. (D) Western blot quantification of NF-κB signaling pathway(F-G)Activation of the IL-17 pathway abolishes the antioxidant effect of Pn@Janus TPP. Data are shown as mean ± SD and analyzed by one-way ANOVA with Tukey's multiple comparisons test. Statistical significance is set as ∗P < 0.05, ∗∗P < 0.01, ∗∗∗P < 0.001,∗∗∗∗P < 0.0001.

At the molecular level, western blot analysis revealed that the nanoplatform significantly downregulates IL-17a expression and potently inhibits NF-κB pathway activation by modulating TRAF6 ubiquitination (Fig. 6A–D). The functional relevance of this mechanism was unequivocally confirmed by rescue experiments: subsequent stimulation with an IL-17 agonist reinstated pathway activity, leading to a surge in intracellular ROS levels and a consequent decline in osteogenic capability (Fig. 6F and G).

### 3.5 In vivo biosafety evaluation

**Fig. 7:**
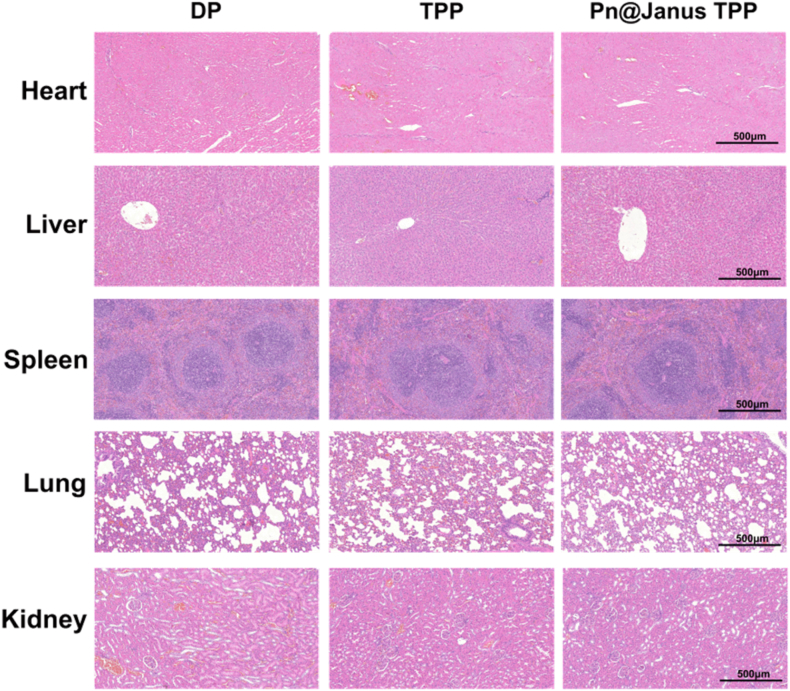
In vivo biosafety evaluation of the Pn@Janus TPP membrane in major organs. H&E staining of the heart, liver, spleen, lung, and kidney tissues harvested 28 days after implantation.

To evaluate the in vivo biocompatibility of Pn@Janus TPP, H&E staining of major organs (heart, liver, spleen, lung, kidney) was performed 28 days after implantation. As shown in Fig. 7, all groups maintained normal histological structures: myocardial fibers were tightly arranged with clear striations; liver lobules were intact with hepatocytes arranged radially around the central vein; splenic lymphoid follicles had a clear structure; alveolar septa in lung tissue remained thin; and glomerular capillary loops in kidney cortex were intact without protein casts in renal tubules. No toxic lesions such as fatty degeneration, vacuolation, or fibrosis were observed, confirming that the membrane did not induce systemic toxicity and met the safety requirements for biomedical materials.

### 3.6 Micro-CT evaluation of alveolar bone regeneration in diabetic periodontitis rat model

**Fig. 8:**
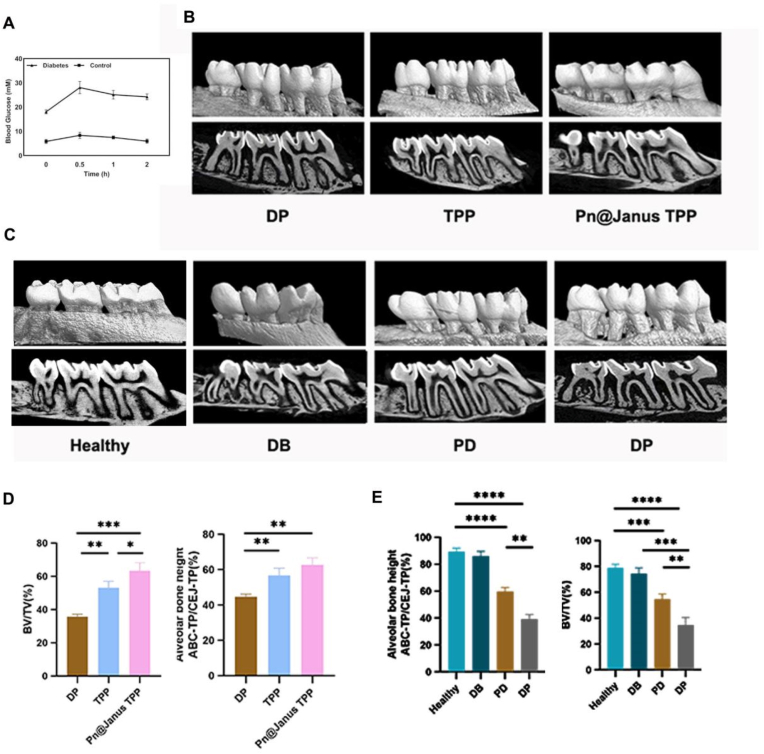
Alveolar bone repair in a rat diabetic periodontitis model. (A)OGTT confirms successful induction of diabetes. (B) Validation of the diabetic periodontitis rat model by Micro-CT 3D reconstruction images. (C)Micro-CT analysis confirms the efficacy of Pn@Janus TPP in promoting alveolar bone regeneration. (D)Statistical analysis of diabetic periodontitis rat model. (E)Statistical analysis of alveolar bone height loss. Data are shown as mean ± SD and analyzed by one-way ANOVA with Tukey's multiple comparisons test. Statistical significance is set as ∗P < 0.05, ∗∗P < 0.01, ∗∗∗P < 0.001,∗∗∗∗P < 0.0001.

For in vivo evaluation of the Pn@Janus TPP nanofibrous barrier membrane, we explored its efficacy in treating diabetes-associated periodontitis using a ligature-induced chronic periodontitis model of maxillae in streptozotocin (STZ)-induced diabetic Sprague-Dawley (SD) rats (Fig. 8A). After the implantation of the membrane, the rats were sacrificed at day 28. Alveolar bone regeneration was visualized using Micro-CT. The extent of alveolar bone loss was quantified by measuring the linear distance ratio between the alveolar bone crest (ABC) to the apex stop (TP) and the cementoenamel junction (CEJ) to the apex stop (ABC-TP/CEJ-TP), as well as the bone volume/tissue volume (BV/TV) percentage at the palatal root furcation of the maxillary second molar. As shown in Fig. 8D, the Healthy group and DB group showed no significant difference in alveolar bone height and BV/TV. In contrast, the PD group exhibited a significant decrease in ABC-TP/CEJ-TP and BV/TV compared to the Healthy group, while the DP group showed a more severe reduction, confirming the synergistic effect of diabetes and periodontitis in exacerbating alveolar bone loss.

After 28 days of implantation, as shown in Fig. 8E, the DP control group still maintained a high level of alveolar bone loss, reflecting the poor self-repair ability of periodontal tissues in the diabetic inflammatory microenvironment. Meanwhile, the Pn@Janus TPP group exhibited a significant restoration. This indicated that Pn@Janus TPP effectively promoted new bone formation and reversed alveolar bone resorption.

### 3.7 Histological evaluation of periodontal regeneration and inflammation in diabetic periodontitis rat model

**Fig. 9:**
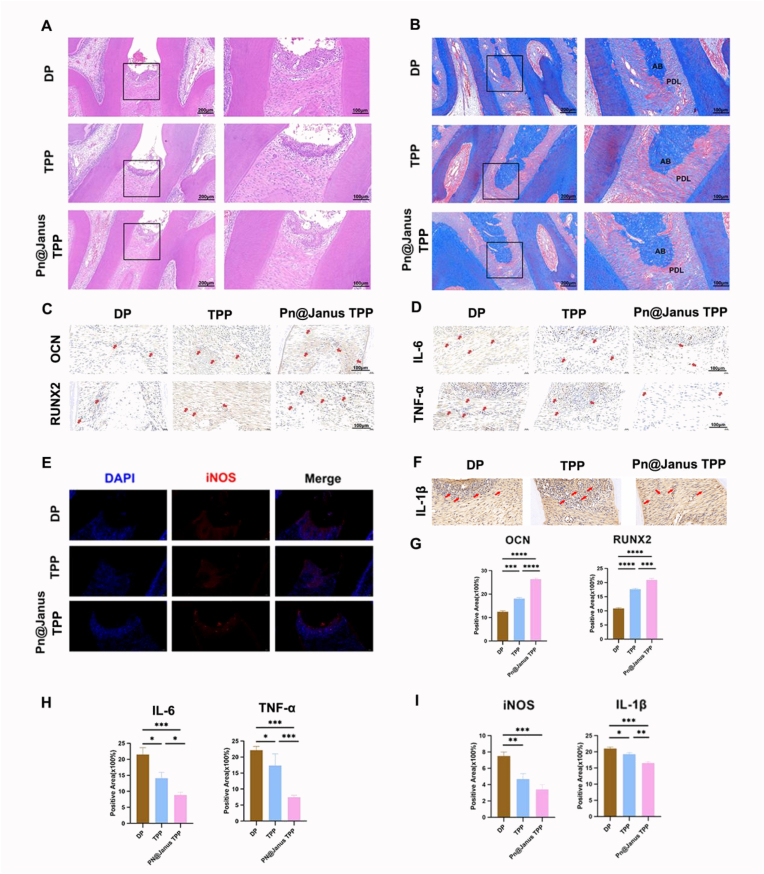
Histology illustration images of the rat maxillary. (A) H&E staining of maxilla tissue section. The black framed area was the enlarged area which was exhibited on the right. (B) Masson trichrome staining of maxilla tissue section. AB represents alveolar bone and PDL represents periodontal ligament. The black framed area was the enlarged area which was exhibited on the right. (C)Immunohistochemistry staining of OCN and RUNX-2. (D)Immunohistochemistry staining of IL-6 and TNF-α. (E)Immunofluorescence staining of INOS. (F)Immunohistochemistry staining of IL-1β. (G-I)Quantitative Analysis. Data are shown as mean ± SD and analyzed by one-way ANOVA with Tukey's multiple comparisons test. Statistical significance is set as ∗P < 0.05, ∗∗P < 0.01, ∗∗∗P < 0.001.

Upon retrieval at day 28, the surgical site in the Pn@Janus TPP group was well-healed with minimal visual signs of inflammation. Fragments of the degrading membrane were still visible in situ, maintaining structural integrity but showing signs of absorption and integration with surrounding soft tissue, consistent with its ongoing degradation profile.Furthermore, histological analyses were performed to evaluate periodontal tissue regeneration and inflammatory infiltration. Hematoxylin and Eosin (H&E) staining showed that the DP control group exhibited severe destruction of gingival epithelial integrity, with disordered polarity of stratified epithelial cells, interrupted basal cell layer, extensive superficial cell necrosis, and diffuse infiltration of neutrophils and lymphocytes in the stroma; periodontal ligament (PDL) fibers were loosely arranged with widened gaps and rootward migration of junctional epithelium (Fig. 9A). In contrast, the Pn@Janus TPP group showed regular arrangement of gingival epithelial cells, clear demarcation between the basal and spinous layers, only scattered inflammatory cells in the stroma, and orderly oblique arrangement of PDL fiber bundles, with new osteoid deposition at the alveolar bone margin forming a tight functional attachment with PDL fibers (Fig. 9B). Masson staining further revealed that the DP control group had disorganized, unevenly thickened new collagen fibers in the PDL region with multiple discontinuities, while the Pn@Janus TPP group exhibited dense, orderly oblique arrangement of PDL principal fibers consistent with the physiological characteristics of Sharpey's fibers, and a gradual transition between the new osteoid area and mature bone matrix at the alveolar bone edge.

Immunohistochemical (IHC) staining was used to detect the expression of osteogenic markers and pro-inflammatory cytokines. As shown in Fig. 9C and G, the Pn@Janus TPP group showed higher expression levels of RUNX2 and OCN compared to the DP control group and TPP group, confirming sustained osteogenic promotion by the membrane. The results demonstrated that the Pn@Janus TPP group reduced the expression of both IL-6 and TNF-α compared to the DP control group (Fig. 9D–H), confirming its potent anti-inflammatory effects. Compared to the DP and TPP groups, Pn@Janus TPP significantly reduced the levels of iNOS and IL-1β (Fig. 9E–I), demonstrating its dual anti-inflammatory effect.

## 4 Discussion

Periodontitis in diabetic patients presents a uniquely challenging microenvironment that undermines conventional regenerative strategies [33]. Hyperglycemia, AGEs, and the resulting chronic inflammation create a self-amplifying loop of oxidative stress and impaired cellular function, rendering passive barrier membranes largely ineffective. This work addresses this clinical problem through the rational design of a Janus-structured biomaterial, Pn@Janus TPP, which integrates spatial guidance with active biofunctionality specifically tailored to disrupt the pathological circuits of diabetic periodontitis.

The design philosophy of Pn@Janus TPP is fundamentally anchored in addressing the specific failures of regeneration under diabetic conditions. The diabetic state impairs fibroblast and osteoblast function while promoting a pro-inflammatory milieu [3,34]. Our Janus architecture directly counteracts this by providing spatially resolved functions. The dense, smooth side acts as a definitive physical barrier, effectively blocking the invasion of HGFs,a key soft tissue cell [35], thereby preventing soft tissue invasion into the bone defect area and creating a closed space for osteogenic regeneration—an essential function of GTR membranes [36]. This ensures the creation and maintenance of a secluded space, a non-negotiable prerequisite for the slower regeneration of the periodontal complex. Conversely, the opposing side was engineered not merely to be porous but to be actively pro-regenerative. The conjugation of a PEG/nHA hydrogel addresses two key diabetic deficiencies: poor cell adhesion due to compromised cellular activity and lack of sustained osteogenic signaling [37,38]. The shift to a hydrophilic surface (62° contact angle) significantly enhanced BMSC attachment and spreading. Moreover, the nano-hydroxyapatite component was incorporated to provide a sustained, local release of calcium ions [39]. This mimics the natural mineralization process and serves as a constant biochemical cue to promote osteogenic differentiation [40], directly countering the inhibitory effects of high glucose and inflammatory cytokines on bone formation.

A central pathological driver in diabetic periodontitis is the excessive generation of ROS, which damages cellular components and disrupts signaling pathways [41,42]. To actively neutralize this threat, we functionalized the membrane with tea polyphenol-reduced graphene oxide (TPG). This component leverages the potent, natural antioxidant properties of tea polyphenols [43,44]. The significant reduction in intracellular ROS levels in BMSCs cultured under diabetic-inflammatory conditions (LPS + AGE) directly validates the material's capacity to mitigate oxidative stress. This is not a secondary feature but a foundational one for enabling regeneration in a diabetic context. By scavenging excess ROS, Pn@Janus TPP helps to preserve mitochondrial function and genomic integrity in progenitor cells, thereby protecting their intrinsic regenerative potential from the damaging diabetic milieu.

The most profound mechanistic insight from this study is the immunomodulatory action of Pn@Janus TPP, achieved through the targeted suppression of the IL-17/TRAF-6/NF-κB signaling pathway. This pathway is a critical nexus linking dysregulated immunity in diabetes to accelerated periodontal breakdown. In hyperglycemic conditions, the differentiation of Th17 cells is promoted, leading to elevated IL-17 levels [45]. IL-17 signaling through its receptor activates the adaptor protein TRAF-6, which in turn triggers the canonical NF-κB pathway [[46], [47], [48]], resulting in the transcriptional upregulation of a cascade of pro-inflammatory mediators [49] (e.g., TNF-α, IL-6, IL-1β, MMPs). Our data demonstrate that Pn@Janus TPP effectively interrupts this cascade. Transcriptomic analysis identified the IL-17 pathway as a major target. Subsequent molecular validation confirmed the downregulation of IL-17a and, crucially, the inhibition of TRAF-6 and NF-κB activation [50,51]. This targeted inhibition explains the coordinated downregulation of multiple inflammatory cytokines observed both in vitro and in vivo. The functional causality was solidified by rescue experiments, where exogenous IL-17 stimulation reversed the membrane's beneficial effects on both ROS reduction and osteogenesis [52,53]. This indicates that the material's immunomodulation is central to its function, shifting the local environment from a catabolic, pro-resorptive state to one that supports anabolic tissue formation.

Regarding the TPG component, its antibacterial activity may involve several aspects: on one hand, the tea polyphenols themselves [54,55], integral to TPG composition, can exert antibacterial effects through membrane disruption, inhibition of key enzymatic activities, and chelation of metal ions essential for bacteria. On the other hand, the graphene-based nanomaterial may also contribute via physical contact-mediated bacterial damage [56,57]. It should be noted that the present study primarily focused on the host immunomodulatory and osteogenic functions of the material. The direct antibacterial efficacy of Pn@Janus TPP against keystone periodontal pathogens such as *P. gingivalis* was not evaluated, representing a limitation of this work. Future studies will specifically investigate the antibacterial and anti-biofilm properties of the material to comprehensively assess its potential in managing the infectious aspect of periodontal regeneration.

The in vivo efficacy of Pn@Janus TPP in a diabetic rat periodontitis model provides integrative validation of this multifunctional design. The significant alveolar bone regeneration, restoration of organized periodontal ligament fibers, and concomitant reduction in inflammatory cell infiltration are the direct, synergistic outcomes of its combined properties. The immunohistochemical findings—showing enhanced osteogenic marker expression alongside suppressed pro-inflammatory cytokine levels—faithfully recapitulate the mechanisms elucidated in vitro. Furthermore, the absence of systemic toxicity confirms the material's biocompatibility and local mode of action, which is essential for clinical translation.While our study focused on local immunomodulation and observed no systemic toxicity within the 28-day period, the potential implications of sustained local IL-17 pathway suppression on systemic immune surveillance, particularly in immunocompromised diabetic hosts, remain an open question. This aspect warrants careful investigation in future long-term studies to fully evaluate the safety profile of localized immunomodulatory biomaterials.

## 5 Conclusion

In conclusion, we have developed a novel dual-functional Janus nanofibrous membrane, Pn@Janus TPP, through rational integration of a dense barrier layer and a bioactive PEG/nHA hydrogel-porous layer. This membrane demonstrated excellent biocompatibility, potent ROS-scavenging ability, and the capacity to significantly promote osteogenic/cementogenic differentiation of BMSCs under diabetic-inflammatory conditions. Mechanistically, its regenerative and anti-inflammatory effects were mediated, at least in part, through the suppression of the IL-17/TRAF-6/NF-κB signaling pathway. In a diabetic periodontitis rat model, Pn@Janus TPP effectively enhanced alveolar bone regeneration and modulated the local inflammatory response without systemic toxicity. This work not only presents Pn@Janus TPP as a promising advanced GTR membrane for diabetic periodontitis but also provides deeper mechanistic insight into biomaterial-mediated immunomodulation for complex tissue regeneration.

## CRediT authorship contribution statement

**Feiyang Wang:** Conceptualization, Data curation, Formal analysis, Funding acquisition, Investigation, Methodology, Project administration, Resources, Software, Supervision, Validation, Visualization, Writing – original draft, Writing – review & editing. **Yue Wang:** Methodology, Project administration, Resources, Software, Supervision, Validation. **Jiaqi Sheng:** Funding acquisition, Investigation, Resources, Software. **Kewei Zhang:** Data curation, Funding acquisition, Investigation, Methodology. **Yu Cao:** Project administration, Software, Validation, Visualization. **Xiang Han:** Software, Supervision, Validation, Visualization, Writing – review & editing. **Ke Yan:** Funding acquisition, Investigation, Methodology, Resources. **Xiaoqian Wang:** Conceptualization, Data curation, Formal analysis, Funding acquisition, Investigation, Methodology, Project administration, Validation, Visualization.

## Declaration of competing interest

The authors declare that they have no known competing financial interests or personal relationships that could have appeared to influence the work reported in this paper.

## Data Availability

Data will be made available on request.
